# Longitudinal Analysis of Obesity Drug Use and Public Awareness

**DOI:** 10.1001/jamanetworkopen.2024.57232

**Published:** 2025-01-29

**Authors:** Philipp Berning, Rishav Adhikari, Adrian E. Schroer, Yara A. Jelwan, Alexander C. Razavi, Michael J. Blaha, Omar Dzaye

**Affiliations:** 1 Johns Hopkins Ciccarone Center for the Prevention of Cardiovascular Disease, Johns Hopkins University School of Medicine, Baltimore, Maryland; 2 Division of Vascular and Interventional Radiology, Department of Radiology, University of Texas Southwestern Medical Center, Dallas, Texas

## Abstract

**Importance:**

Obesity, a chronic disease with escalating global prevalence, poses considerable health risks. Glucagon-like peptide 1 receptor agonists (GLP-1RAs), including liraglutide, semaglutide, and tirzepatide, have demonstrated efficacy for weight loss in clinical trials. The paradigm shift in the approach to obesity management drugs (OMDs) may offer an opportunity to examine online search activity and prescription trends.

**Objective:**

To investigate prescription patterns and online search trends for OMD and correlating usage dynamics with public engagement regarding these medications.

**Design, Setting, and Participants:**

This repeated cross-sectional study analyzed online searches and corresponding data for dispensed prescriptions derived from the IQVIA National Prescription Audit, a comprehensive audit capturing approximately 90% of US retail prescription dispensing that is then projected to population-level data. Data from online searches and on prescriptions from the National Prescription Audit for US Food and Drug Administration–approved OMDs were collected from July 1, 2017, through February 29, 2024. This population-level analysis included all eligible individuals using either the Google search engine or receiving prescriptions.

**Exposures:**

US monthly query fractions as online searches per 10 million total searches and monthly prescriptions dispensed for OMDs.

**Main Outcome and Measures:**

Trends in OMD prescriptions and online searches were visually analyzed, and quantitative correlations were calculated using Spearman rank correlation.

**Results:**

During the study period, 69 213 936 prescriptions for OMDs were dispensed in the US, with an overall increase of 0.76 to 0.80 million from July 2017 to June 2018 and 1.29 to 1.51 million from March 2023 to February 2024 and a mean (SD) annual growth rate of 5.3% (9.4%). Total monthly OMD prescriptions reached 1.5 million in February 2024, accounting for 0.41% of all prescriptions that month. Phentermine, semaglutide (Wegovy; Novo Nordisk), liraglutide (Saxenda; Novo Nordisk), and tirzepatide (Zepbound; Eli Lilly) were most prescribed. By February 2024, phentermine had approximately 0.74 million monthly prescriptions, Wegovy had 0.42 million, and Zepbound had 0.25 million. Most prescriptions were issued by advanced practice practitioners (APP) and primary care physicians (PCPs) and internists. The APPs’ share increased from 25.3% in July 2017 to 40.6% in February 2024, while PCPs’ and internists’ share decreased from 57.9% to 48.1%. Wegovy’s share of total OMDs increased to 27.7% and 28.3% and Zepbound’s share to 16.1% and 16.5% (both February 2024) among APPs and PCPs and internists, respectively. Phentermine’s dropped from 86.3% and 85.1% (July 2017) to 50.8% and 49.7% (February 2024), respectively. Online searches per 10 million reflected prescription trends, with Wegovy at 636.3, Zepbound at 468.9, and phentermine at 301.8 (all February 2024). The strongest correlations between prescriptions and search volumes were observed for semaglutide (Wegovy; *r* = 0.97) and tirzepatide (Zepbound; *r* = 0.90).

**Conclusions and Relevance:**

This repeated cross-sectional study shows the dynamic growth and evolving share of new obesity medications since 2017 and a significant shift toward semaglutide and tirzepatide prescribing. The joint surge in prescriptions and online searches and their close correlation highlight the real-time associations between public awareness and prescription trends.

## Key Points

**Question:**

What are the trends in obesity management drug (OMD) dispensed prescriptions and their correlation with public online search interest?

**Findings:**

This cross-sectional study using a repeated approach and including 69 213 936 OMD dispensed prescriptions revealed an increase from 0.76 million in July 2017 to 1.51 million in February 2024, with an upward trend in monthly phentermine and glucagon-like peptide 1 receptor agonist prescriptions. There was a robust positive correlation between public online search activity for semaglutide (Wegovy; *r* = 0.97) and tirzepatide (Zepbound; *r* = 0.90) and their prescription trends.

**Meaning:**

These findings suggest that surveillance of dispensed prescriptions of OMD through US online search behavior may be a powerful tool to gauge interest and forecast future use.

## Teaser

This cross-sectional study investigates US prescription patterns and public online search trends for obesity management drugs.

## Introduction

Obesity is increasingly recognized as a chronic disease that may lead to numerous and severe health complications, including type 2 diabetes, cardiovascular disease, chronic kidney disease, hepatic steatosis, and certain types of cancer.^1^ The prevalence of obesity, defined as body mass index (calculated as weight in kilograms divided by height in meters squared) of at least 30, has reached alarming levels globally within the past decades.^2^ For the US, age-adjusted prevalence of obesity has increased from 30.5% in 1999-2000 to 42.4% in 2017-2018.^3^ Even more striking, the prevalence of very severe obesity (defined as BMI ≥40) has markedly increased from 4.7% to 9.2% during these periods.^3^ Several analyses underscore that obesity-related conditions are a major contributor to all-cause mortality and increasing health care costs.^4^

Amid escalating efforts to combat the obesity epidemic, pharmaceutical interventions have become essential components of obesity treatment. Traditional approaches, such as adopting a healthy diet and engaging in regular physical activity, have demonstrated limitations in achieving sustained weight loss and reducing associated comorbidities such as type 2 diabetes, hypertension, dyslipidemia, and cardiovascular disease.^5,6,7,8^ While older therapies such as phentermine have limited efficacy, newer obesity management drugs (OMDs) have demonstrated robust efficacy in clinical trials, offering new therapeutic options for treating obesity.^9^ In particular, the glucagon-like peptide 1 receptor agonists (GLP-1RAs) approved for weight loss, including liraglutide (Saxenda; Novo Nordisk) in 2014 and semaglutide (Wegovy; Novo Nordisk) in 2021, have emerged as a highly effective drug class. They promote satiety, reduce appetite, and regulate blood glucose levels.^10,11,12^ Semaglutide demonstrated significant efficacy in several clinical trials, with studies showing clinically relevant mean weight losses of approximately 15% of body weight.^13,14,15,16^ Furthermore, the recent US Food and Drug Administration (FDA) approval of the dual GLP-1 and gastric inhibitory polypeptide agonist tirzepatide (Zepbound; Eli Lilly) for weight management in November 2023 further expands the armamentarium of incretin-based weight loss therapies.^11,15,17^ Public awareness of OMDs, particularly semaglutide and its brand names, has surged across various platforms.^18^ The noteworthy surge in demand has resulted in shortages of semaglutide and its brands in 2023, leading to concerning supply issues for patients with diabetes who rely on GLP-1RA therapy.^19^

In the context of this unique confluence of market forces centered on OMDs, including rapid scientific innovation, extensive media attention, and pronounced product shortages, we sought to characterize the association between public interest and trends in dispensed prescriptions of OMDs. The potential correlation of prescription trends and public interest may have implications for forecasting and anticipating the complex dynamics of OMDs in coming years.

## Methods

In this repeated cross-sectional study, prescription data were gathered from July 1, 2017, through February 29, 2024, from the IQVIA National Prescription Audit (NPA) and an online search engine (Google) for US Food and Drug Administration (FDA)–approved OMDs. The study did not include individual patient information or data; therefore, no ethics committee approval or informed consent were required according to the Common Rule. The reporting of results adheres to the Strengthening the Reporting of Observational Studies in Epidemiology (STROBE) guideline for cross-sectional studies.

### Data Collection

#### Drug Prescription Data

Drug prescription data were extracted from the NPA, which provides a measure of overall national prescription dispensing information from retail, mail-order, and long-term care pharmacies. These prescription data include approximately 90% of all outpatient prescription activity for dispensed medications in the US and are then projected to estimate 100% of retail transactions. Prescriptions from all payers, including Medicare and Medicaid, are captured. Of note, however, our dataset is influenced by Medicare policies that did not cover obesity medications during the study period, while self-pay or out-of-pocket prescriptions, including those of Medicare beneficiaries, were included. Further detailed information on the data collection process has been previously published.^20^ IQVIA links the NPA to the American Medical Association’s Physician Masterfile and other professional organization records to confirm the primary specialty of prescribers. Accordingly, due to the nature of the dataset, this study includes only dispensed prescriptions; prescribed but not dispensed medications were unable to be included. Hereafter, the term prescriptions refers exclusively to dispensed prescriptions.

We queried monthly dispensed prescriptions for FDA-approved drug brands for weight loss and obesity management (eTable 1 in Supplement 1), as well as approved GLP-1RAs for diabetes management with known weight loss effects (eTable 2 in Supplement 1). The starting point for this study period in 2017 was selected to account for data availability in IQVIA and the initial approval of semaglutide, which, although initially approved for diabetes, provides a relevant baseline for observing subsequent drug uptake and usage patterns of this therapeutic class in obesity management. From the NPA, we extracted monthly dispensed prescriptions, prescriber specialty, and brand names of the drugs used among all patients. Our initial analyses encompassed total, new, and refill prescriptions, with each set of analyses yielding substantively similar findings.

Regarding prescriber information, we categorized physician assistants and nurse practitioners as advanced practice practitioners (APPs). General practitioners are referred to as primary care physicians (PCPs) and internists, which includes family practice, general practice, general preventive medicine, geriatrics, internal medicine, internal medicine and pediatrics, osteopathic medicine, and pediatrics, as previously reported.^21,22^

The queried drugs were categorized based on brand names as (1) FDA-approved OMDs (naltrexone and bupropion [Contrave; Orexigen Therapeutics], orlistat [Alli (GlaxoSmithKline), Orlistat (generic), Xenical (Roche)], phentermine [Lomaira (KVK-Tech), Phentermine (generic), Qsymia (Vivus)], semaglutide [Wegovy], liraglutide [Saxenda], and tirzepatide [Zepbound]) and (2) GLP-1RAs with weight loss effects (tirzepatide [Mounjaro; Eli Lilly], semaglutide [Ozempic (Novo Nordisk), Rybelsus (Novo Nordisk)], and liraglutide [Victoza; Novo Nordisk]). For the visualization of prescription trends by substance name, prescription data were gathered for liraglutide (Saxenda, Victoza), semaglutide (Ozempic, Rybelsus, Wegovy), and tirzepatide (Mounjaro, Zepbound).

#### Online Search Activity

Online search data for the US were retrieved for the period between July 1, 2017, and February 29, 2024, using the Google Health Trends Application Programming Interface, as described in previous studies.^23,24,25^ Google Health Trends captures a sample of all public Google searches, including data from both signed-out and some signed-in users. While it excludes infrequent searches and duplicate queries from the same user within a short time frame, Google Health Trends cannot entirely exclude the possibility that the same user may use multiple devices. Online search data were extracted from Google Health Trends as query fractions per 10 million searches representative for approximately 89% of all online searches for all indicated drug brand names (eTables 3 and 4 in Supplement 1) as well as for chemical drug names (liraglutide, semaglutide, tirzepatide) (eTable 4 in Supplement 1). To ensure specificity and accuracy in capturing actual drug searches, we only accounted for correct brand and/or chemical drugs names.

### Statistical Analysis

Calculations and data visualization, including Spearman rank correlation between monthly prescriptions and online searches, were performed using Excel, version 16.91 (Microsoft Corporation) and R, version 4.1.1 (R Foundation). Calculations of relative changes were performed using the respective difference between prescription and online search measurement and the previous value, with the previous value as the denominator. Annual change rates were calculated as mean annual percent changes using log transformation. For data visualization, a simple moving average (SMA) model was applied to calculate the mean values of prescriptions and/or searches over specified monthly periods. This method was used to smooth out short-term fluctuations, reduce data volatility, and enhance the visibility of mid- and long-term trends in the shown datasets. The SMA model facilitated a clearer interpretation of the underlying patterns in prescription rates and search volumes, allowing for more accurate trend analysis. The SMA calculations were performed using the tidyquant package in R.

## Results

### Prescription Trends for OMDs

During the study period, 69 213 936 prescriptions for OMDs were dispensed in the US (Table). Prescriptions for OMDs showed a steady increase from 0.76 to 0.80 million in the first 12 months (July 2017 to June 2018) to 1.29 to 1.51 million in the final 12 months (March 2023 to February 2024). The mean (SD) annual (12-month) growth rate over this span reached 5.3% (9.4%) for FDA-approved OMDs, underscoring a sustained upward trajectory.

**Table.** Annual Prescription Trends for OMDs and GLP-1RAs With Weight Loss Effects Between 2018 and 2023, IQVIA National Prescription Audit (January 2018 to December 2023)

Year	No. of prescriptions (annual percent change)			
	OMDs^a^	Semaglutide (Wegovy, Ozempic, Rybelsus)	Tirzepatide (Mounjaro, Zepbound)	GLP-1RAs with weight loss effects^b^
2018	9 060 679	347 128	0	4 599 064
2019	8 926 721 (−1.5)	2 192 863 (531.7)	0	6 160 831 (34.0)
2020	8 688 979 (−2.7)	4 883 081 (122.7)	0	8 375 377 (35.9)
2021	9 654 487 (11.1)	8 630 164 (76.7)	0	11 259 455 (34.4)
2022	10 337 271 (7.1)	15 147 512 (75.5)	2 482 961^c^ (June 2022-December 2022)	19 113 488 (69.8)
2023	15 164 706 (46.7)	29 931 933 (97.6)	5 696 709 (January 2023-July 2023) (129.4)^c^; 11 035 526 (January 2023-December 2023)	37 968 969 (98.7)

Abbreviations: GLP-1RA, glucagon-like peptide 1 receptor agonist; OMD, obesity management drug.

^a^ US Food and Drug Administration–approved OMDs are naltrexone and bupropion (Contrave), orlistat (Alli, Orlistat, Xenical), phentermine (Lomaira, Phentermine, Qsymia), semaglutide (Wegovy), liraglutide (Saxenda), and tirzepatide (Zepbound).

^b^ The GLP-1RAs with weight loss effects are tirzepatide (Mounjaro), semaglutide (Ozempic, Rybelsus), and liraglutide (Victoza).

^c^ The first reported prescriptions recorded in June 2022 followed Mounjaro’s approval in May 2022. For comparison, we analyzed the periods from May 2022 to December 2022 (7 months) and January 2023 to July 2023 (7 months).

**Figure 1.**
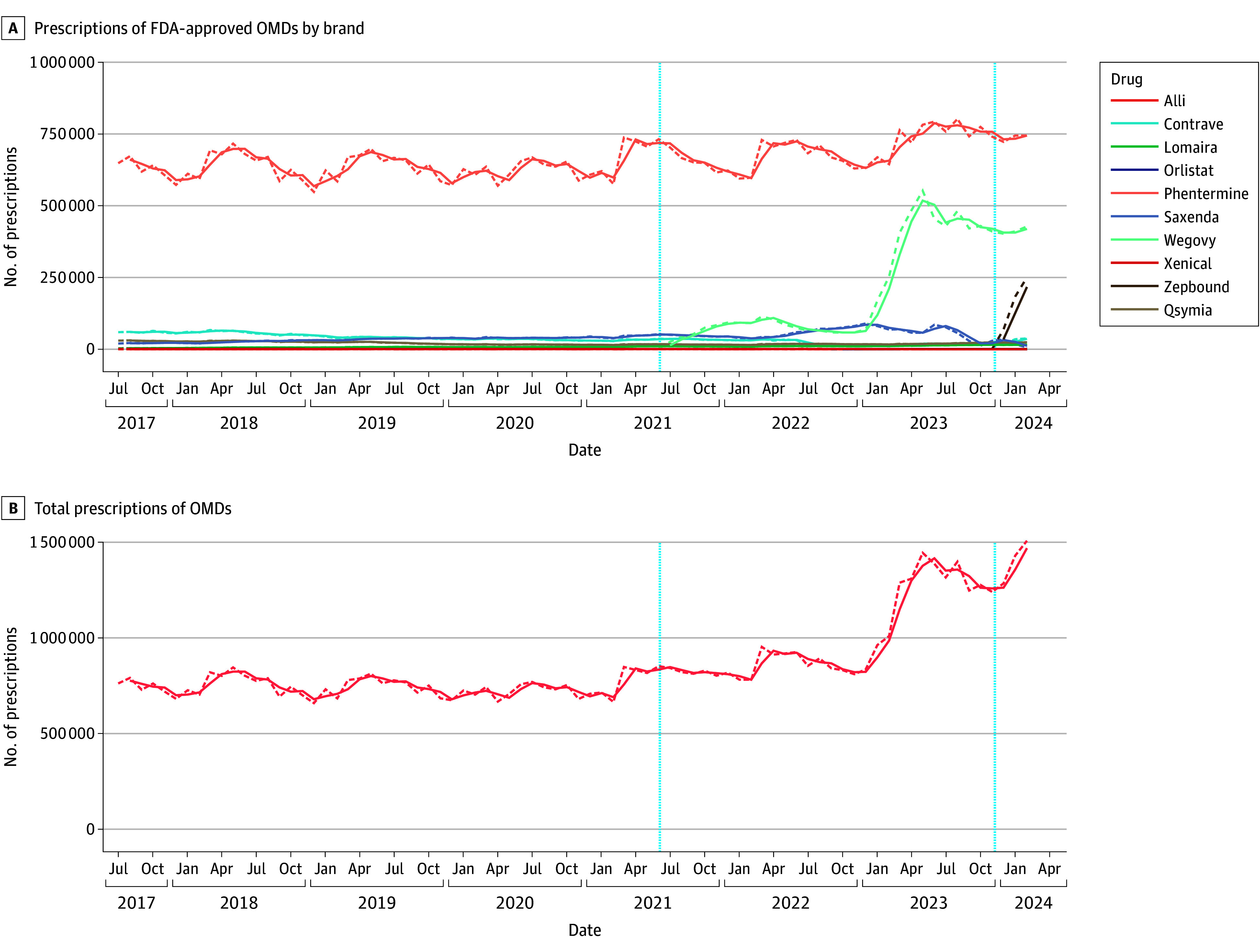
Prescription Trends for Obesity Management Drugs (OMDs), IQVIA National Prescription Audit (July 2017-February 2024) The dashed curves indicate the monthly trends and the solid curves, the 2-month moving average for each drug. The vertical dashed line at June 2021 indicates Wegovy approval for weight loss and that at November 2023, Zepbound approval for weight loss. FDA indicates US Food and Drug Administration.

Next, we evaluated prescription trends across all medical specialties for OMD brands (Figure 1). The highest prescription activity was observed for phentermine, semaglutide (Wegovy), tirzepatide (Zepbound), all displaying an upward trend over the study duration (Figure 1A). Notably, by February 2024, phentermine had reached approximately 0.74 million monthly prescriptions. Dispensed prescriptions for Wegovy (approved June 2021) and Zepbound (approved November 2023) reached 0.42 million and 0.25 million monthly prescriptions by February 2024, respectively. Phentermine prescriptions exhibited seasonal peaks each year during the study period, with the highest annual numbers occurring in quarters 2 to 3. Conversely, other OMDs showed a declining trend between July 2017 and February 2024, with monthly prescriptions falling below 0.02 million since October 2023 (Saxenda) or July 2022 (Contrave), while other brands showed only minimal usage (Alli, Lomaira, Orlistat, Xenical), with less than 0.02 million monthly prescriptions throughout the study period. As shown in Figure 1B, aggregated prescription data for OMDs revealed an increasing trend reaching 1.51 million monthly prescriptions in February 2024. The share of OMDs in the landscape of all dispensed prescriptions exhibited an upward trend. Starting at 0.21% in July 2017, shares steadily increased over the study period to a maximum of 0.41% of all prescriptions in the US by February 2024 (eFigure 1 in Supplement 1).

**Figure 2.**
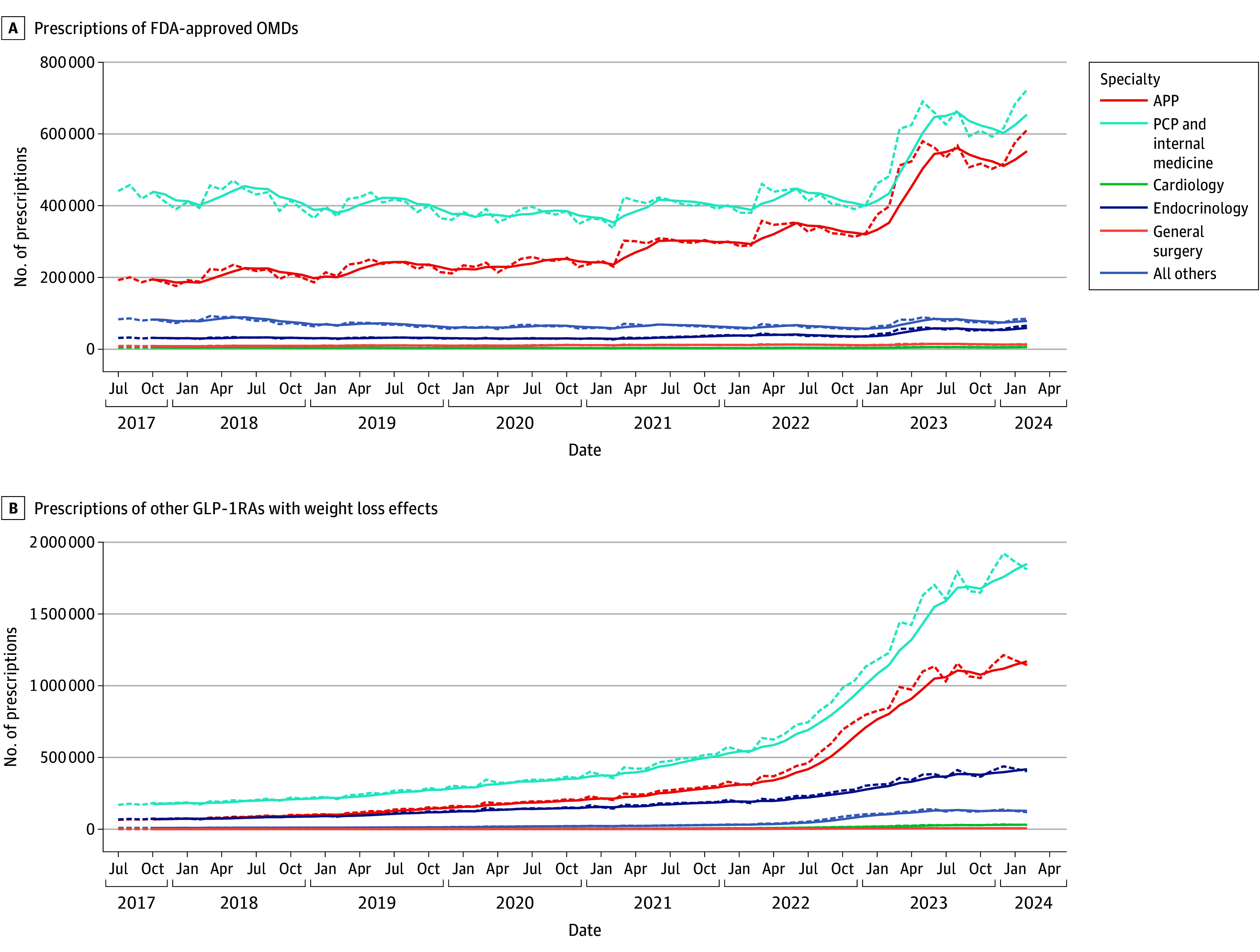
Prescribing of Obesity Management Drugs (OMDs) Across Specialties, IQVIA National Prescription Audit (July 2017-February 2024) The dashed curves indicate the monthly trends and the solid curves, the 2-month moving average for each drug. APP indicates advanced practice practitioner; FDA, US Food and Drug Administration; GLP-1RA, glucagon-like peptide 1 receptor agonist; PCP, primary care physician.

Most prescriptions for OMDs were issued by APPs and PCPs or internists as opposed to cardiologists, endocrinologists, general surgeons, and all other specialists (Figure 2). At the beginning of the study period (July 2017), the national distribution of OMD prescriptions across specialties showed APPs at 25.3%, PCPs and internists at 57.9%, endocrinologists at 4.1%, general surgeons at 1.2%, cardiologists at 0.6%, and all other specialists at 10.9%. However, by February 2024, the most recent time point, a notable shift in prescription patterns was noted. APPs increased their share to 40.6%, while PCPs and internists decreased to 48.1%. Other specialties also showed slight decreases or no changes: endocrinology to 4.4%, general surgery to 0.9%, cardiology to 0.5%, and all other specialties to 5.6%. These data revealed a substantial increase in the proportion of OMD prescriptions by APPs, while other specialists, particularly PCPs and internists, experienced a decrease in their share of prescriptions.

We further evaluated OMD choices among APPs and PCPs and internists as the main prescribing specialists. Specifically, the proportion of Wegovy in OMD prescriptions increased substantially, reaching a share of 27.7% and 28.3% for APPs and PCPs and internists, respectively, by February 2024 (eFigure 2 in Supplement 1). Additionally, by February 2024, Zepbound reached a share of 16.1% and 16.5% among APPs and PCPs and internists, respectively. In contrast, phentermine prescribing decreased, accounting for 50.8% and 49.7% of the OMD share for APPs and PCPs and internists in February 2024 compared with 86.3% and 85.1% in July 2017, respectively (eFigure 2 in Supplement 1). Of note, the drug choices for both APPs and PCPs and internists across available OMDs were nearly identical, underscoring a consistent preference across these medical specialists.

### Online Search Activity for Weight Loss Drugs

**Figure 3.**
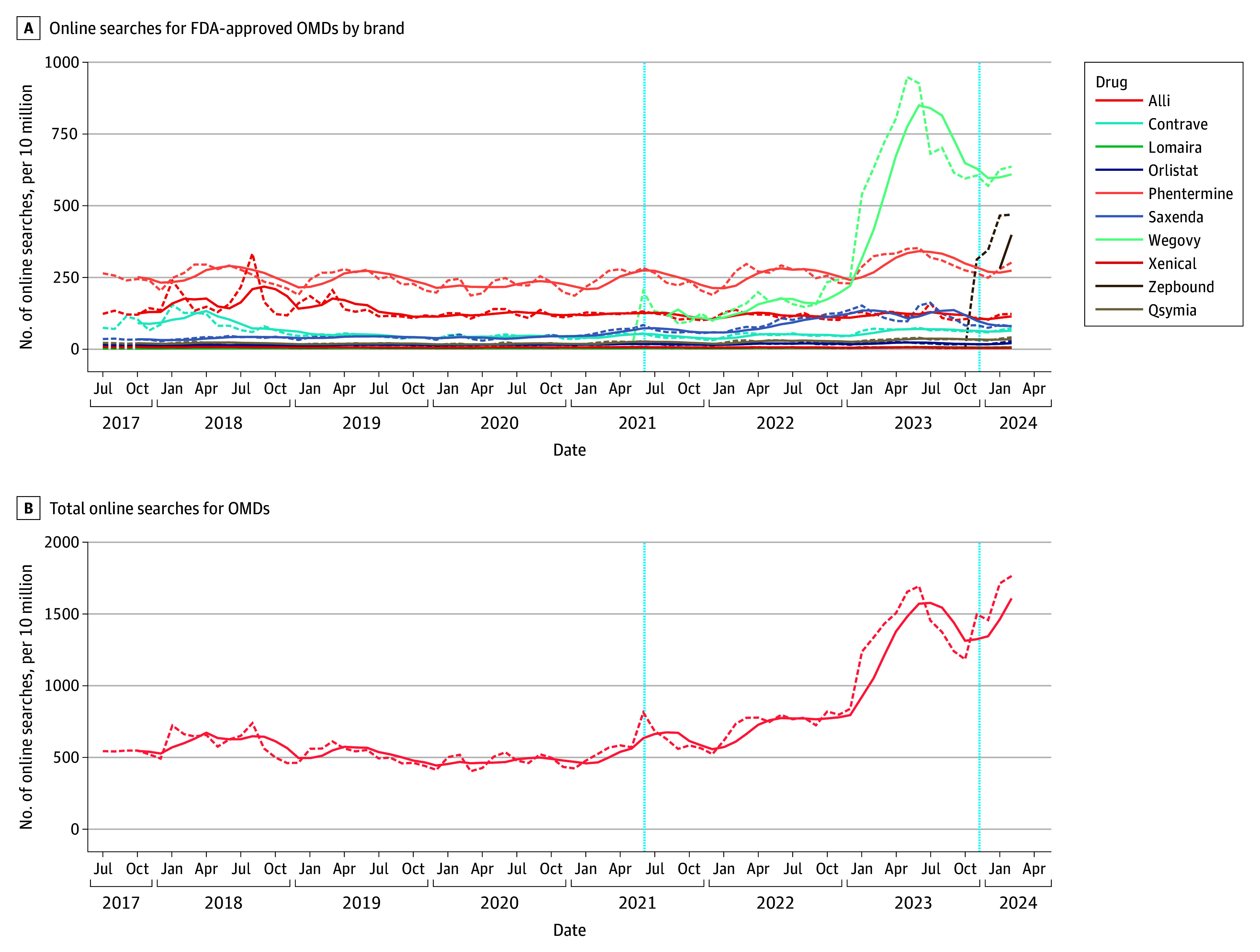
Online Searches for Obesity Management Drugs (OMDs), IQVIA National Prescription Audit (July 2017-February 2024) Dashed curves indicate the monthly trends and solid curves, the 4-month moving average for each drug. The vertical dashed line at June 2021 indicates Wegovy approval for weight loss and that at November 2023, Zepbound approval for weight loss. FDA indicates US Food and Drug Administration.

Next, we queried online search volumes for the respective OMDs throughout the study period. Among the approved OMDs, Wegovy emerged with the highest online search volume of 636.3 per 10 million searches, followed by Zepbound of 468.9 and phentermine of 301.8 per 10 million searches as of February 2024 (Figure 3A). Most prominently, Zepbound showed an increase from 312.7 to 468.9 per 10 million searches and Wegovy an increase from 202.9 to 636.3 per 10 million searches since FDA approval. For Wegovy, we observed a peak in online search activity in May 2023 with 948.2 per 10 million searches, which was followed by a decline of approximately 40.0% by December 2023, after which time an upward trend was noted again. Similar to prescription trends, online search activity showed an increasing trend of aggregated online volumes for OMDs of 543.9 per 10 million searches in July 2017 to a maximum in February 2024 of 1679.2 per 10 million searches (Figure 3B).

### Prescriptions and Searches for Liraglutide, Semaglutide, and Tirzepatide

**Figure 4.**
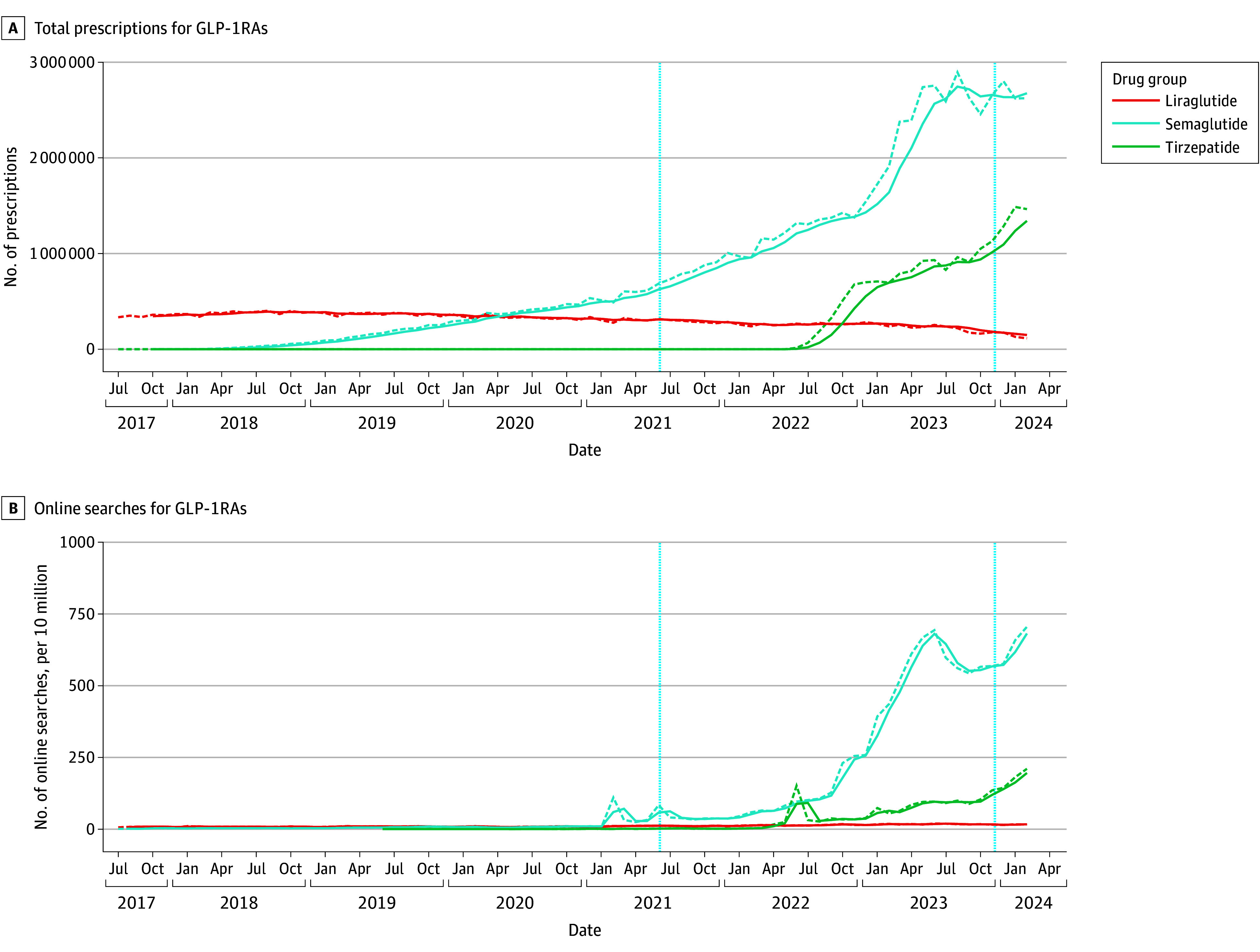
Prescription and Online Search Trends for Glucagon-Like Peptide 1 Receptor Agonists (GLP-1RAs), IQVIA National Prescription Audit (July 2017-February 2024) Data are presented for all brands of liraglutide (Saxenda, Victoza), semaglutide (Ozempic, Rybelsus, Wegovy), and tirzepatide (Mounjaro, Zepbound). Dashed curves indicate the monthly trends and solid curves, the 2-month (prescriptions) or 4-month (online searches) moving average for each drug group. The vertical dashed line at June 2021 indicates Wegovy approval for weight loss and that at November 2023, Zepbound approval for weight loss.

To account for potential off-label applications of GLP-1RAs that are not primarily approved for obesity management but share the same chemical drug under different brand names and labels, we analyzed prescription and online search trends by chemical name for liraglutide, semaglutide, and tirzepatide. As shown in Figure 4A, increasing prescription rates were noted for semaglutide, with 2.6 million prescriptions in February 2024, an increase of 1.9 million since the approval of Wegovy as an OMD in June 2021 (0.7 million prescriptions). Similarly, tirzepatide showed a clear increase after its initial approval in May 2022 for type 2 diabetes. After receiving the OMD label (Zepbound) in November 2023, tirzepatide prescriptions increased from 1.1 million to 1.4 million monthly prescriptions by February 2024, while liraglutide showed a decline from 0.33 million (July 2017) to 0.11 million (February 2024) monthly prescriptions. Accordingly, online search data were queried for the chemical drug names as depicted in Figure 4B. In line with the corresponding prescription data, the highest overall online search activity was observed for semaglutide, followed by tirzepatide and liraglutide with 705.3, 210.5, and 17.0 searches per 10 million, respectively, in February 2024.

### Prescriptions and Searches for GLP-1RA Without an Obesity Label

We further elucidated longitudinal trends for the following GLP-1RA brands that, while active in weight management, do not possess an OMD label: Ozempic (semaglutide), Rybelsus (semaglutide), Mounjaro (tirzepatide), and Victoza (liraglutide). In total, 96 598 054 prescriptions were issued for these drugs throughout the study period (eFigure 1 in Supplement 1), reflecting an increase from 0.3 million prescriptions in July 2017 to 3.5 million prescriptions in February 2024 and a mean (SD) annual growth rate of 38.5% (31.7%). Ozempic, initially approved for type 2 diabetes in December 2017 and later receiving additional labeling for cardiovascular risk reduction for patients with type 2 diabetes in January 2020,^26^ showed a substantial surge in monthly prescriptions, reaching a peak of 2.2 million in August 2023, followed by 2.0 million in February 2024 (eFigure 3 in Supplement 1). Similarly, for Mounjaro, introduced in May 2022, monthly prescriptions showed a surge, increasing from 0.01 million in June 2022 to 1.2 million in February 2024. Additionally, increasing search activity for the study period was observed for Ozempic, Mounjaro, Rybelsus, and Victoza with 2031.7, 653.1, 88.3, and 57.1 per 10 million searches, respectively, as well as aggregated data with 2830.3 per 10 million searches as of February 2024 (eFigure 4 in Supplement 1).

Drug choices among both APPs and PCPs and internists for these drugs are shown in eFigure 5 in Supplement 1. In July 2017, before the approval of the other drugs, virtually all prescriptions were issued for Victoza. In February 2024, Ozempic, Mounjaro, and Rybelsus accounted for 53.7%, 36.8%, and 6.1% of all APP prescriptions, respectively (eFigure 5A in Supplement 1), reflecting an increase in their proportions. In contrast, Victoza prescriptions showed a decline, reaching 3.3% of all APP prescriptions. Comparable trends were observed for PCP and internist prescriptions for these drugs (eFigure 5B in Supplement 1).

### Correlations Between Prescriptions and Online Searches

In a next step, we analyzed correlation patterns between prescriptions and online searches throughout the study period for selected GLP-1RAs with obesity management applications and highest prescription rates and online search activity. eFigure 6 in Supplement 1 shows a correlation matrix with a size- and color-coded representation of significant correlation coefficients between prescription and online search activity for phentermine, Saxenda, Wegovy, Ozempic, Zepbound, and Mounjaro. For these brands, the correlation coefficients between prescriptions and online searches were 0.78 (95% CI, 0.68-0.86; *P* < .001) for phentermine, 0.67 (95% CI, 0.52-0.78; *P* < .001) for Saxenda, 0.97 (95% CI, 0.96-0.98; *P* < .001) for Wegovy, 0.99 (95% CI, 0.98-0.99; *P* < .001) for Ozempic, 0.90 (95% CI, 0.84-0.94; *P* < .001) for Zepbound, and 0.97 (95% CI, 0.95-0.98; *P* < .001) for Mounjaro.

## Discussion

This repeated cross-sectional study provides a detailed population-level overview of prescription data and online search trends for obesity medications. Given the complexity of current prescribing practices, it also considers additional GLP-1RAs potentially used off label for obesity management in clinical practice.

Our study had 3 main findings. First, over the more than 7-year study period, OMDs showed a 1.7-fold increase with a 5.3% annual growth rate, accounting for 0.41% of all US prescriptions by February 2024. Phentermine, semaglutide, and tirzepatide were the most prescribed drugs for obesity treatment. Second, most prescriptions for obesity management were issued by APPs and PCPs or internists, with APPs showing a steadily increasing share that reached 40.6% of obesity drugs, while PCP and internists’ share of prescriptions decreased from 58.0% to 48.1% throughout the study period. Notably, no differences were noted in prescription behaviors across the main prescribing specialties. Third, Wegovy had the highest online search interest among OMDs, followed by Zepbound, whereas phentermine search trends remained almost unchanged over time. High correlations between prescriptions and online searches for OMDs were observed for semaglutide (Wegovy) and tirzepatide (Zepbound). These findings suggest that online search activity mirrors prescription trends, reflecting the growing usage of these medications in obesity management.

The observed findings in prescription activity may be explained by the results from major clinical trials. Despite no direct comparisons being available, semaglutide (Wegovy) is widely regarded as a more effective weight loss agent than prior generations of OMDs, such as liraglutide and phentermine.^16,27,28^ Additionally, even more recently, tirzepatide, a dual gastric inhibitory polypeptide and GLP-1RA, has been shown to be highly effective in obesity management in large clinical trials, which led to its approval in November 2023.^12,17,29^ With weekly injectable GLP-1RA representing the most effective drug class among OMDs, the rapid rise in online awareness, as well as prescription volumes of semaglutide and tirzepatide, compared with the stagnant trends for phentermine and phentermine-containing formulations (Qsymia), may be a reflection of their superior efficacy. Another factor contributing to the increasing use of GLP-1RAs in obesity treatment may be explained by their favorable safety profile. While GLP-1RAs, including semaglutide and tirzepatide, are generally well tolerated with common but mild gastrointestinal side effects, phentermine can cause potentially more serious side effects, such as anxiety, insomnia, and high blood pressure.^16,30,31^ The observed seasonal peaks in phentermine prescriptions, in contrast to the lack of similar trends in other OMDs, particularly GLP-1RAs, may be associated with differences in their growth dynamics. While prescriptions patterns for phentermine appeared to show recurrent patterns with peaks in quarters 2 to 3 each year, newer OMDs, such as GLP-1RAs are experiencing rapid growth and may not yet have reached stable prescription rates. As such, the continuous upward trajectory in their usage could mask any underlying seasonal variations that might become more apparent once growth stabilizes.

In terms of prescriber specialties, our data show that most prescriptions were issued by APPs and PCP or internists, which aligns with previous analyses that found similar prescription patterns for the use of GLP-1RAs in diabetes and cardiovascular disease treatment.^21,32^ Notably, no difference was observed in the adoption of newer OMDs, such as Wegovy and Zepbound, when comparing nonphysician specialists (APPs) with PCPs and internists. The GLP-1RAs initially approved for diabetes management, such as semaglutide (Ozempic, Rybelsus) and tirzepatide (Mounjaro), are often prescribed off label for obesity treatment. We considered these applications in our analysis, recognizing that the antiobesity effects of these medications may be beneficial for both diabetes management and obesity treatment.^33^ This dual benefit highlights the versatility of GLP-1RAs in addressing both metabolic disorders.

The reported shortages in supplies of Wegovy in 2022 and 2023^19,34^ may explain the observed temporarily declining prescription trends, which were paralleled by decreased online search activity. Supply shortages for obesity-labeled semaglutide (Wegovy) persisted throughout 2023,^19,34,35^ while diabetes-labeled semaglutide (Ozempic) prescription numbers continued to steadily increase based on our observation. This pattern may suggest that Ozempic, although only labeled for diabetes treatment, may have been used in part to meet the demand for antiobesity medication. The increasing prescription numbers for Ozempic paralleled by a decrease in those for Wegovy point to Ozempic’s possible dual role in addressing both diabetes management and weight loss needs, further underscoring the growing recognition of GLP-1RAs’ efficacy in treating obesity, even when primarily approved for diabetes treatment. Our data revealed a sharp increase in prescriptions for Zepbound (tirzepatide),^11^ an FDA-approved OMD as of November 2023. This drug, which shares a nearly identical application mode with its antidiabetic counterpart Mounjaro,^11,12,15,36^ quickly reached more than 16% of all OMD prescriptions. Based on clinical evidence suggesting tirzepatide’s greater efficacy in obesity management in the context of type 2 diabetes compared with semaglutide,^37^ as well as its potential to positively influence obesity-related conditions such as metabolic dysfunction–associated steatohepatitis,^38^ we anticipate that it may become the leading choice in OMD prescriptions.

Online search volume analyses revealed notable trends in public interest for OMDs and GLP-1-RAs without an obesity label. In particular, searches for Wegovy showed a 4.7-fold increase following its FDA approval for obesity treatment. The fluctuations in search activity for Wegovy, including a peak in 2023 followed by a decline, correlating closely with a similar rise and then fall of prescription volumes, may reflect initial excitement, supply constraints, and shifts in public attention. Zepbound’s rise to the second most searched OMD may signal public interest in new obesity treatment options, particularly in the context of product shortages for semaglutide-containing medications. Strong correlations between online searches and prescriptions were observed for semaglutide and tirzepatide brands in contrast to drugs with stable or declining trends (phentermine and liraglutide). These correlations align with our previous reports suggesting that online search activity most accurately reflects drugs with increasing usage, as seen with sodium-glucose cotransporter 2 inhibitors and hepatocellular carcinoma medications.^32,39^ This study’s findings suggest that online search data may serve as an additional, readily available tool for health care systems and pharmaceutical companies to anticipate and prepare for increased demand in OMD supply.

### Limitations

Our study has several limitations. First, prescription data for total generated prescription numbers were considered, which include both newly prescribed drugs and refills or continuation of existing medications with a new prescription number. Therefore, our data analyses might be less sensitive to trends in newly initiated prescriptions, which might thus be underrepresented. Second, this study included only dispensed prescriptions, excluding those that were prescribed but not filled. However, this focus on actual dispensation may also be a strength, as it provides a more accurate reflection of actual medication usage patterns. However, this method ensures that the recorded prescriptions included the majority of patients receiving the drugs, providing a comprehensive overview of the prescription landscape. Third, we primarily examined trends in prescription patterns and compared individual OMDs and did not conduct a comprehensive market analysis. The available data do not permit accounting for factors such as prescription duration, clinical indications, or dosing regimens. Fourth, our metric for public interest, Google Health Trends online search data, solely represents searches on Google and affiliated services, notably excluding searches on social media platforms and traditional media (television, newspapers, magazines, etc). In this context, it is important to consider that public awareness and media coverage may influence consumer behavior and search trends, particularly with the conflation of Ozempic and Wegovy for weight loss. Finally, the identity of individuals conducting online searches (patients, clinicians, media, etc) is unknown.

## Conclusions

This repeated cross-sectional study that examined trends in drug usage and online searches from 2017 to 2024 revealed a dynamic shift in obesity medication patterns, with semaglutide and tirzepatide gaining a significant share of all prescriptions. The parallel surge in prescriptions and online searches, and their strong correlation, highlight the association between internet search activity and clinical adoption. These findings may provide insight for health care professionals and policy makers, as they highlight the rapid adoption by clinicians (including nonphysician professions) of state-of-the-art obesity treatments and their growing public interest. Our data provide a foundation to guide epidemiologic trends and harness real-time search patterns to estimate future trends in the adoption of obesity treatment.

## Notes

Supplement 1. **eFigure 1.** Prescriptions and Share of Obesity Management Drugs and GLP-1RAs Without Obesity Label **eFigure 2.** Obesity Management Drug Choice Among APPs and PCPs/Internists **eFigure 3.** Prescription Trends for GLP-1RAs Without Obesity Label **eFigure 4.** Online Searches for Additional GLP-1RA Weight Loss Effects **eFigure 5.** Choice of Additional GLP-1RAs With Weight Loss Effects Among APPs and PCPs/Internists **eFigure 6.** Correlations Between Prescriptions and Online Searches **eTable 1.** Monthly Prescriptions of Obesity Management Drugs **eTable 2.** Monthly Prescriptions of GLP-1RAs With Weight Loss Effects **eTable 3.** Monthly Online Searches (per 10 Million Searches) for Obesity Management Drugs **eTable 4.** Monthly Online Searches (per 10 Million Searches) for GLP-1RAs With Weight Loss Effects and Drug Names

Supplement 2.
Data Sharing Statement
