# Supplementary material for: Longitudinal Analysis of Obesity Drug Use and Public Awareness
Source: JAMA Netw Open. 2025 Jan 29;8(1):e2457232. doi: 10.1001/jamanetworkopen.2024.57232 (PMC11780480; doi:10.1001/jamanetworkopen.2024.57232)
Supplement: Supplement 1. — eFigure 1. Prescriptions and Share of Obesity Management Drugs and GLP-1RAs Without Obesity Label eFigure 2. Obesity Management Drug Choice Among APPs and PCPs/Internists eFigure 3. Prescription Trends for GLP-1RAs Without Obesity Label eFigure 4. Online Searches for Additional GLP-1RA Weight Loss Effects eFigure 5. Choice of Additional GLP-1RAs With Weight Loss Effects Among APPs and PCPs/Internists eFigure 6. Correlations Between Prescriptions and Online Searches eTable 1. Monthly Prescriptions of Obesity Management Drugs eTable 2. Monthly Prescriptions of GLP-1RAs With Weight Loss Effects eTable 3. Monthly Online Searches (per 10 Million Searches) for Obesity Management Drugs eTable 4. Monthly Online Searches (per 10 Million Searches) for GLP-1RAs With Weight Loss Effects and Drug Names [file jamanetwopen-e2457232-s001.pdf]

Supplemental Online Content

Berning P, Adhikari R, Schroer AE, et al. Longitudinal analysis of obesity drug use and public awareness. *JAMA Netw Open*. 2025;8(1):e2457232. doi:10.1001/jamanetworkopen.2024.57232

eFigure 1. Prescriptions and Share of Obesity Management Drugs and GLP-1RAs Without Obesity Label

eFigure 2. Obesity Management Drug Choice Among APPs and PCPs/Internists

eFigure 3. Prescription Trends for GLP-1RAs Without Obesity Label

eFigure 4. Online Searches for Additional GLP-1RA Weight Loss Effects

eFigure 5. Choice of Additional GLP-1RAs With Weight Loss Effects Among APPs and PCPs/Internists

eFigure 6. Correlations Between Prescriptions and Online Searches

eTable 1. Monthly Prescriptions of Obesity Management Drugs

eTable 2. Monthly Prescriptions of GLP-1RAs With Weight Loss Effects

eTable 3. Monthly Online Searches (per 10 Million Searches) for Obesity Management Drugs

eTable 4. Monthly Online Searches (per 10 Million Searches) for GLP-1RAs With Weight Loss Effects and Drug Names

This supplemental material has been provided by the authors to give readers additional information about their work.

© 2025 Berning P et al. *JAMA Network Open*.

eFigure 1. Prescriptions and Share of Obesity Management Drugs and GLP-1RAs Without Obesity Label

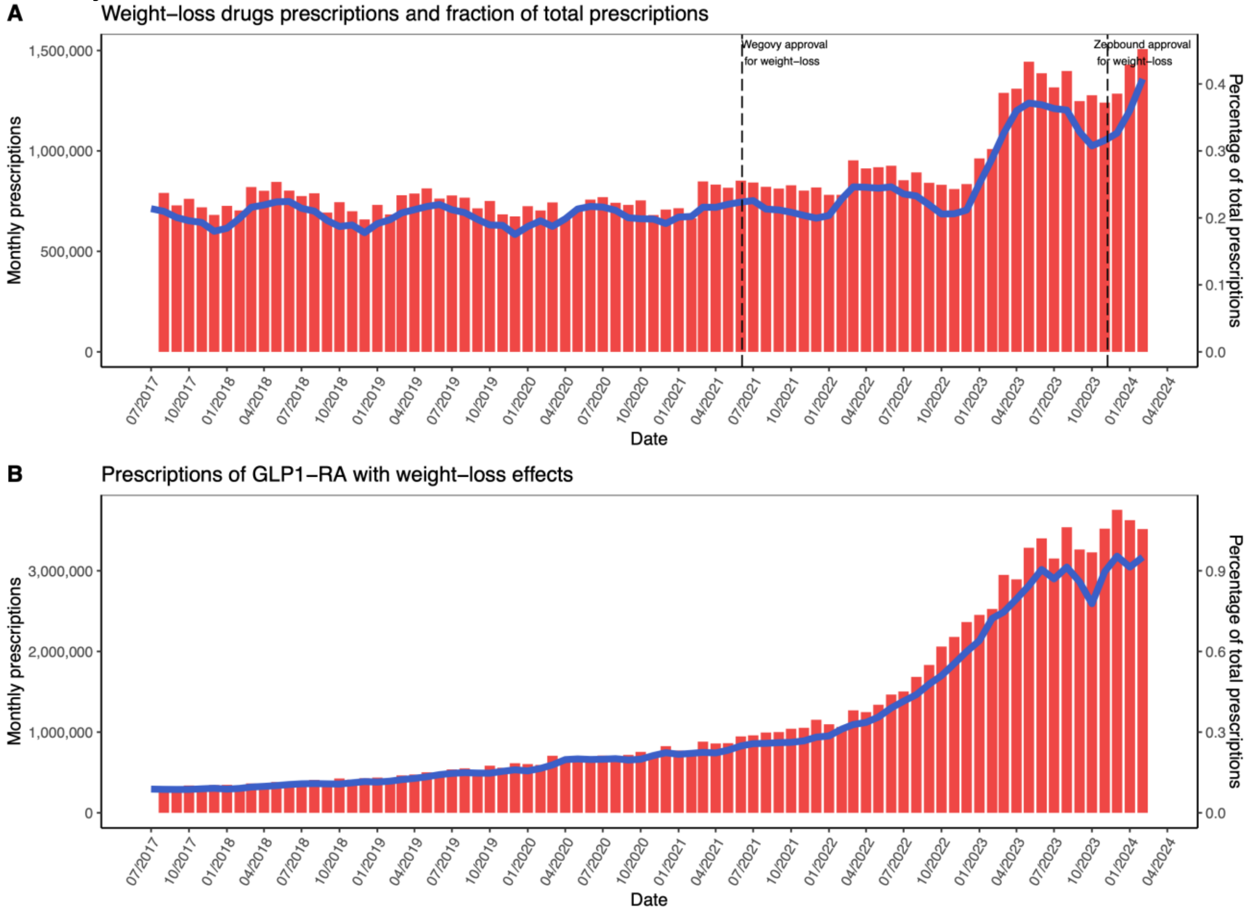

Prescriptions trends and share in total dispensed prescriptions for (A) FDA-approved obesity management drug brands and (B) additional GLP-1 RA with weight-loss effects between 07/2017 and 02/2024. total prescriptions of obesity management drugs between 07/2017 and 02/2024. Bar columns represent the monthly prescription trends; bold curves indicate the 2-month moving average for the percentage of total dispensed prescriptions.
Source: IQVIA National Prescription Audit; July 2017 to February 2024.

© 2025 Berning P et al. *JAMA Network Open*.

eFigure 2. Obesity Management Drug Choice Among APPs and PCPs/Internists

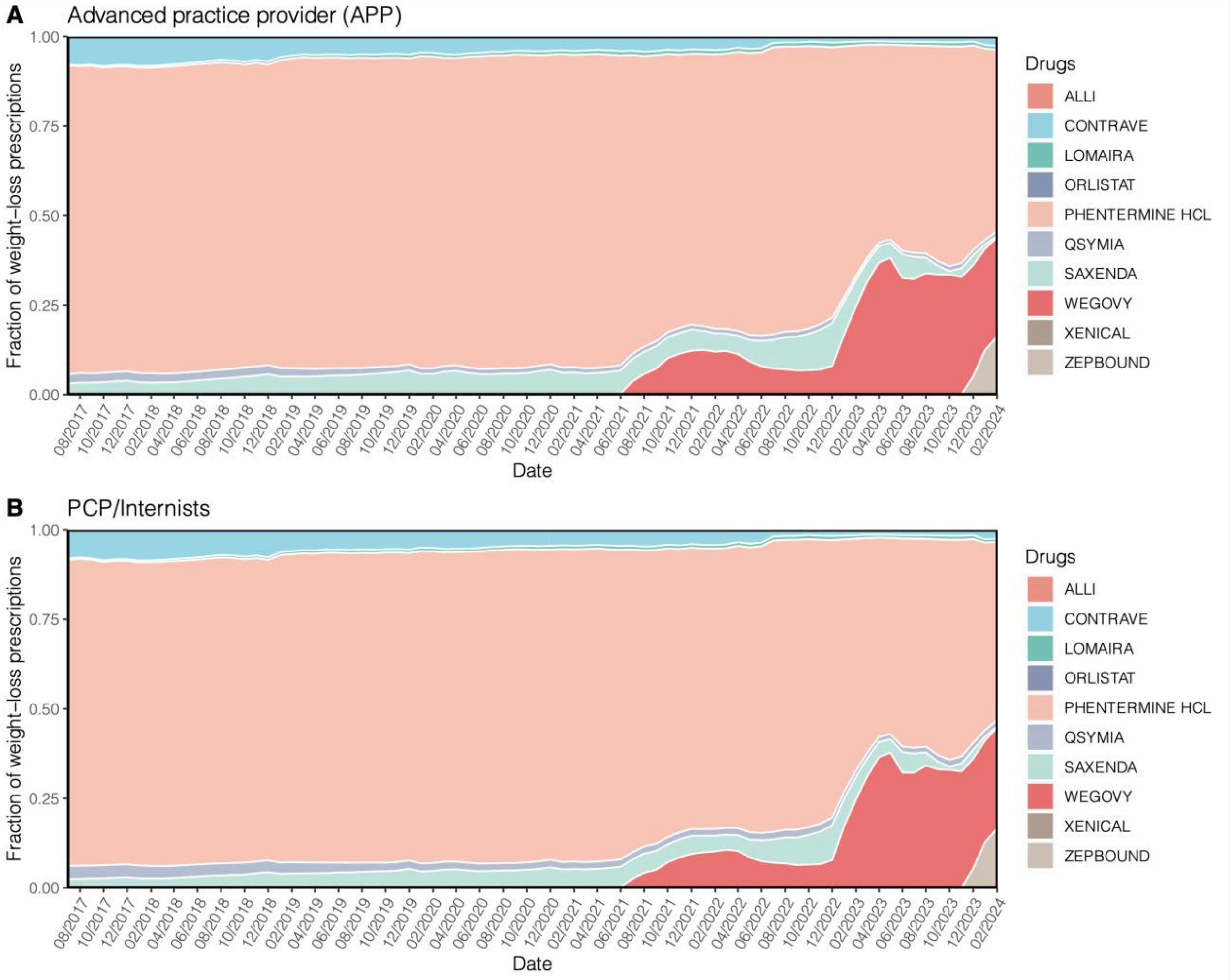

Proportion of monthly dispensed prescriptions for obesity management drugs from (A) advanced practice providers and (B) primary care physicians (PCP)/internists. Data are shown in % of the aggregated total prescription volume for obesity management drugs between 07/2017 and 02/2024.
Source: IQVIA National Prescription Audit; July 2017 to February 2024.

© 2025 Berning P et al. *JAMA Network Open*.

eFigure 3. Prescription Trends for GLP-1RAs Without Obesity Label

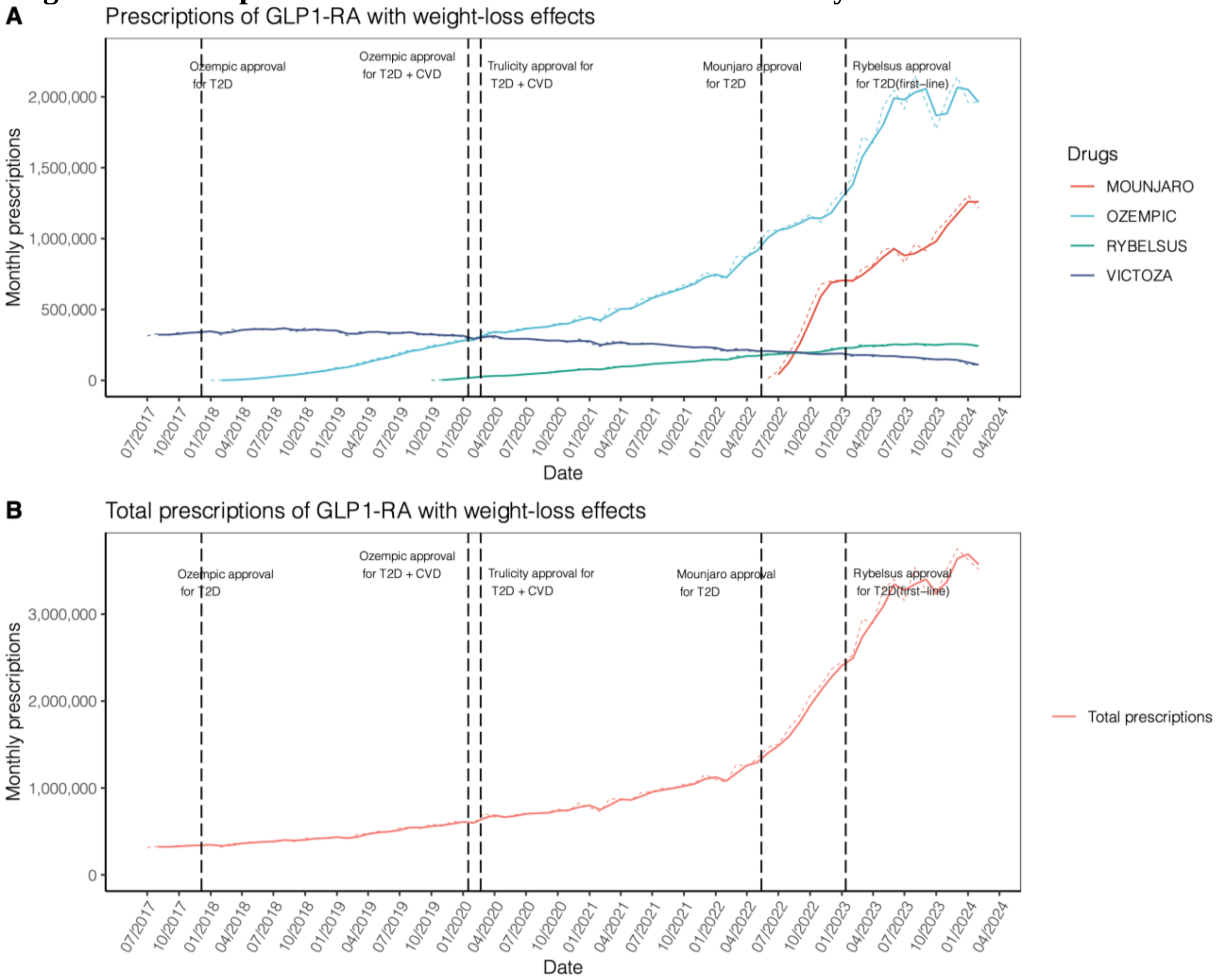

Prescriptions trends for (A) additional GLP-1 RA with weight-loss effects and (B) total prescriptions additional GLP-1 RA with weight-loss effects between 07/2017 and 02/2024. Dashed curves represent the monthly trends; bold curves indicate the 2-month moving average for each drug.
Source: IQVIA National Prescription Audit; July 2017 to February 2024.

© 2025 Berning P et al. *JAMA Network Open*.

eFigure 4. Online Searches for Additional GLP-1RA Weight Loss Effects

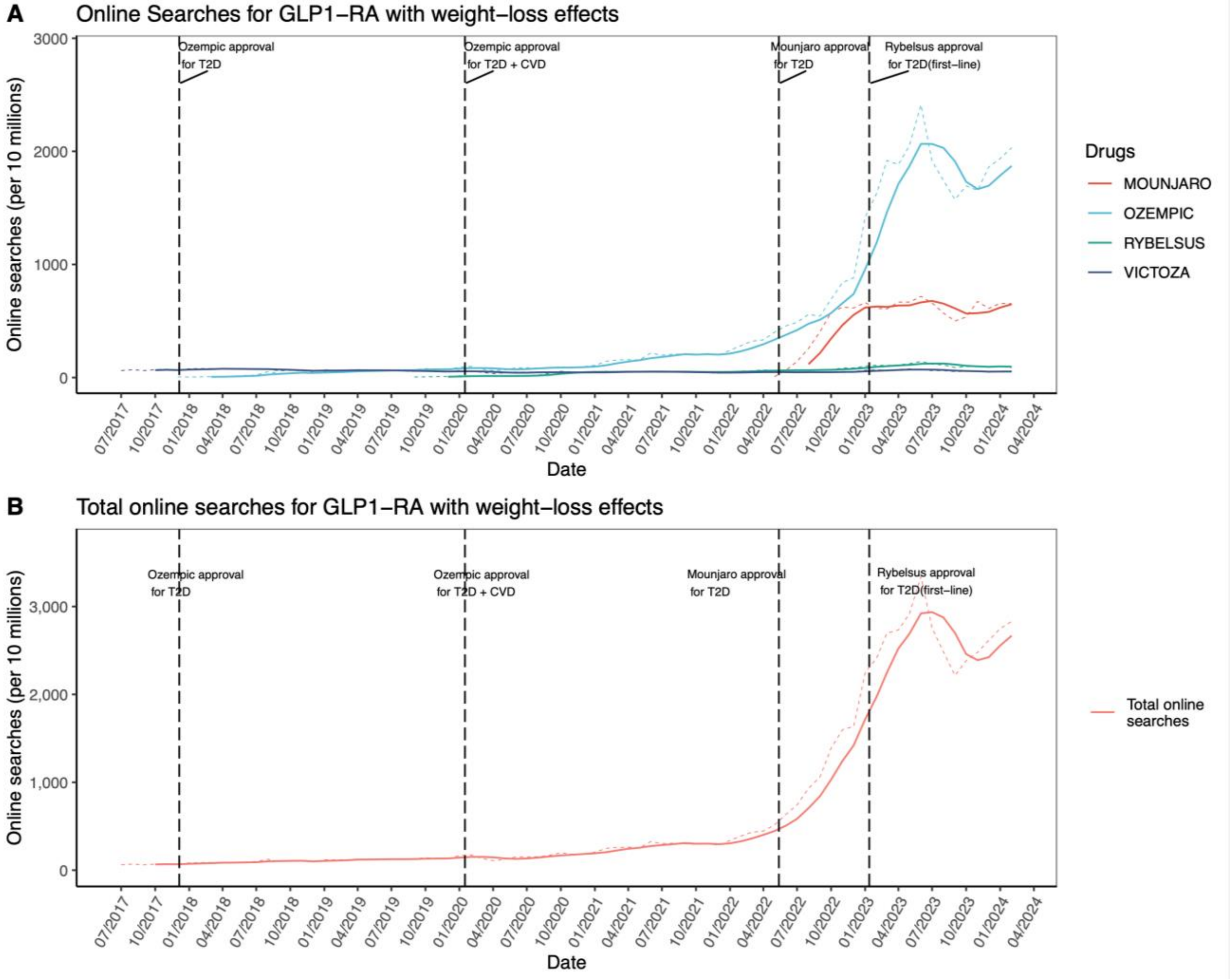

Online Searches for (A) additional GLP-1 RA with weight-loss effects and (B) total online searches for additional GLP-1 RA with weight-loss effects between 07/2017 and 02/2024. Dashed curves represent the monthly trends, bold lines indicate the 4-month moving average for each drug.
Source: IQVIA National Prescription Audit; July 2017 to February 2024.

© 2025 Berning P et al. *JAMA Network Open*.

eFigure 5. Choice of Additional GLP-1RAs With Weight Loss Effects Among APPs and PCPs/Internists

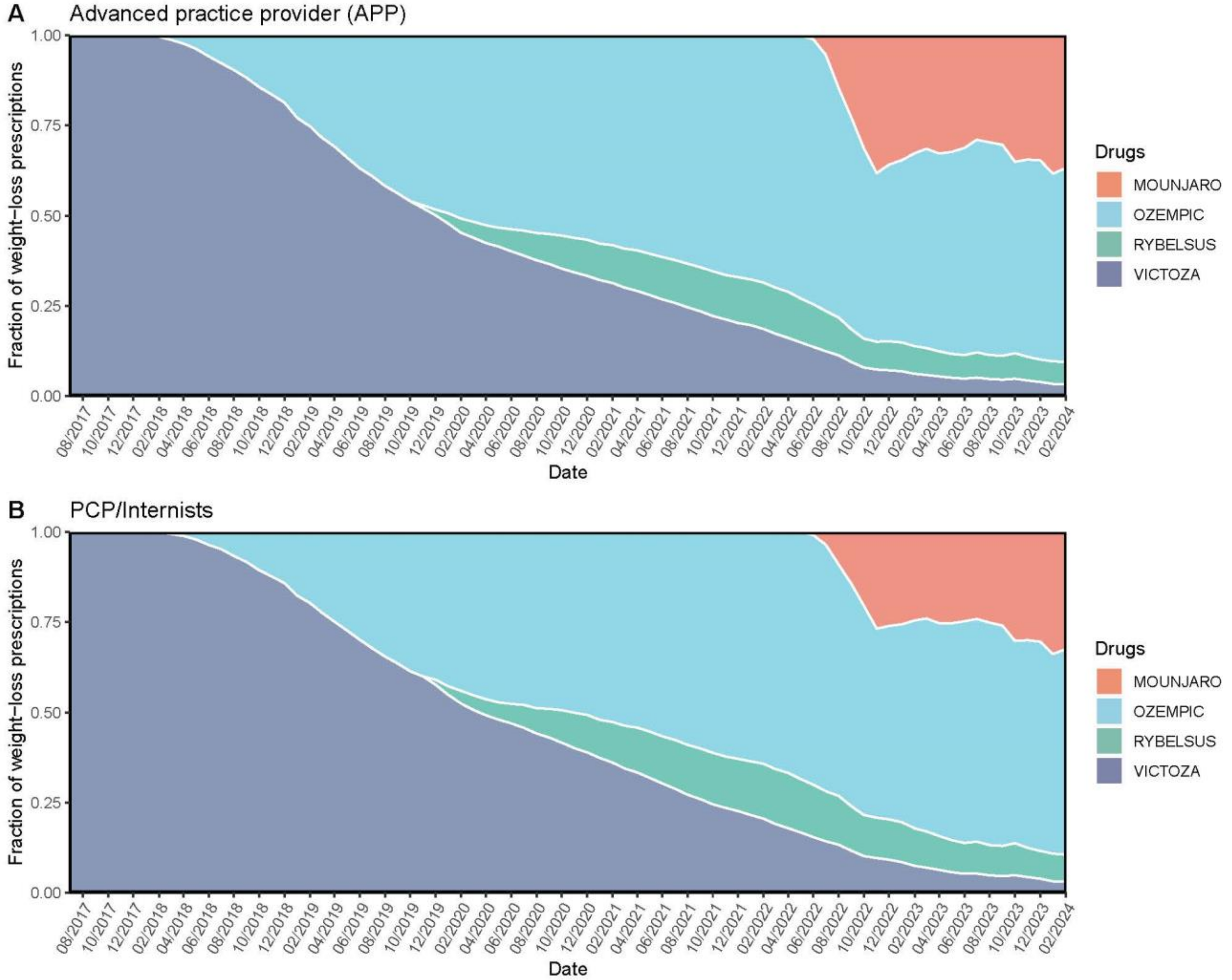

Proportion of monthly dispensed prescriptions for additional GLP-1 RA with weight-loss effects from (A) advanced practice providers and (B) primary care physicians (PCP)/internists. Data are shown in % of the aggregated total prescription volume for obesity management drugs between 07/2017 and 02/2024.
Source: IQVIA National Prescription Audit; July 2017 to February 2024.

© 2025 Berning P et al. *JAMA Network Open*.

eFigure 6. Correlations Between Prescriptions and Online Searches

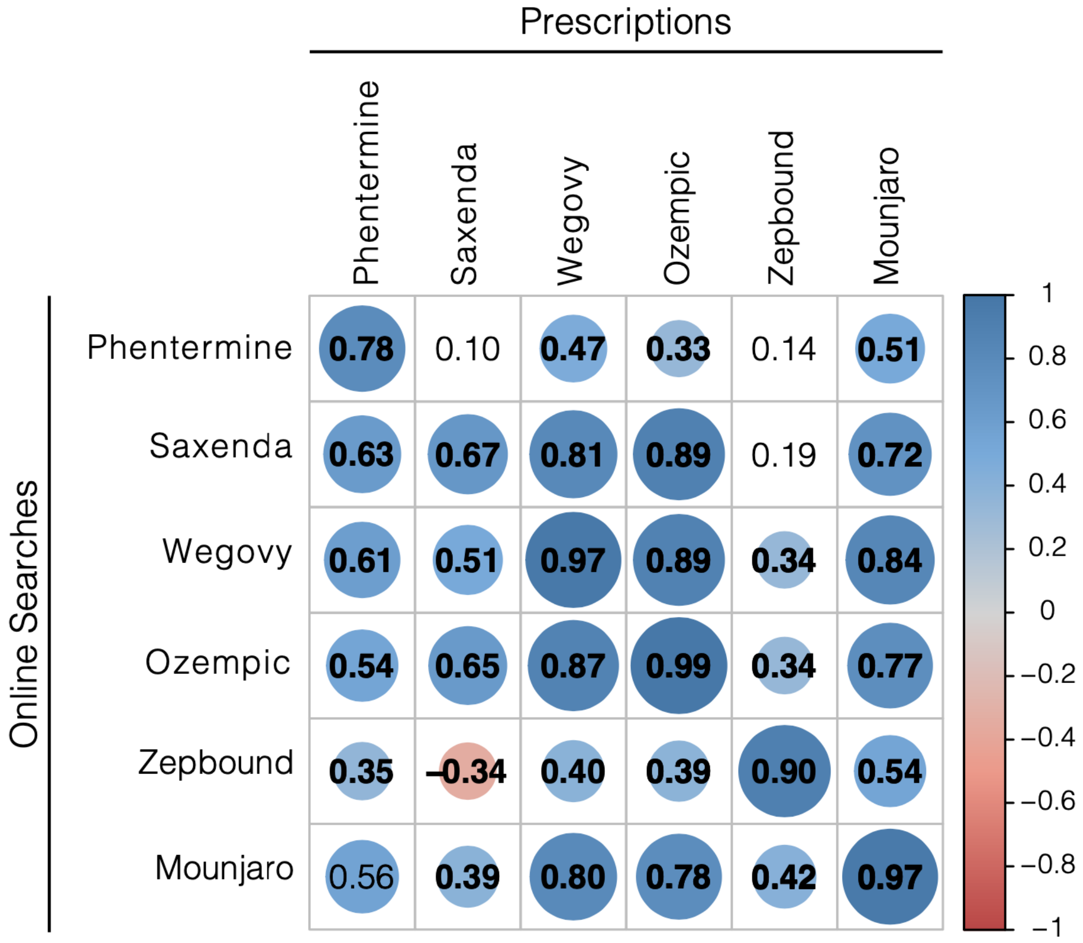

Correlation matrix depicts Spearman's correlation coefficients between prescriptions and online searches for selected drug names (generic name for phentermine) between 07/2017 and 02/2024. Significant values are highlighted in bold, with circle areas representing the absolute values of the corresponding correlation coefficients. Source: IQVIA National Prescription Audit; July 2017 to February 2024.

© 2025 Berning P et al. *JAMA Network Open*.

eTable 1. Monthly Prescriptions of Obesity Management Drugs

Date	ALLI	CONTRAVE	LOMAIRA	ORLISTAT	PHENTERMINE	SAXENDA	WEGOVY	XENICAL	ZEPBOUND	QSYMIA
Jul-17	267	59,421	3,161		648,229	19,715		1,149		29,929
Aug-17	330	60,449	3,479		672,653	21,707		1,283		30,968
Sep-17	303	57,054	3,287		618,802	20,284		1,204		27,916
Oct-17	278	64,101	3,871		640,703	22,068		1,189		29,339
Nov-17	257	57,700	4,040		606,098	22,236		1,122		27,714
Dec-17	258	54,910	4,034		572,597	22,083		1,103		26,395
Jan-18	304	60,689	4,467		611,410	21,048		1,048		27,502
Feb-18	318	58,406	4,465		593,295	20,283		835		26,078
Mar-18	274	66,963	5,322		693,081	23,740		1,025		29,705
Apr-18	244	62,958	5,437		679,201	23,910		977		28,738
May-18	309	64,587	5,993		716,399	26,997		1,021		30,308
Jun-18	291	59,079	5,677		680,321	27,320		1,051		28,583
Jul-18	226	54,725	5,879		657,416	28,196		1,035		27,833
Aug-18	257	53,216	6,212		670,023	29,984		1,034		28,369
Sep-18	286	47,790	5,798		585,822	27,566		973		24,907
Oct-18	327	53,608	6,235		624,883	31,502		1,007		27,170
Nov-18	333	48,040	5,836		588,208	31,255		1,000		25,051
Dec-18	293	48,364	5,807		548,120	31,626		906		23,902
Jan-19	344	43,837	6,620		622,965	31,427		818		25,215
Feb-19	317	38,429	6,249		584,800	29,845		838		23,341
Mar-19	386	41,902	7,172		668,936	34,322		833		25,791
Apr-19	364	43,064	7,484		675,505	35,321		794		25,285
May-19	416	42,838	7,845		698,314	37,473		807		25,583
Jun-19	445	39,833	7,558		655,224	36,489		658		22,490
Jul-19	473	42,096	8,024		667,001	37,415		609		22,611
Aug-19	437	39,847	8,303		658,604	38,287		466		21,322
Sep-19	393	37,962	7,636		612,017	36,691		595		18,950
Oct-19	416	38,507	8,108		643,676	40,020		757		19,265
Nov-19	332	35,096	7,533		585,216	37,804		704		17,398
Dec-19	335	35,861	7,417		571,902	40,533		743		17,182
Jan-20	370	34,413	7,815		627,292	37,109		730		17,162
Feb-20	399	34,155	7,707		607,731	36,464		694		16,379
Mar-20	328	38,853	7,840		636,071	42,266		735		17,450
Apr-20	403	34,923	6,826		569,782	38,997		674		15,397

© 2025 Berning P et al. *JAMA Network Open*.

May-20	437	35,185	7,233		608,702	38,660		730	15,972
Jun-20	478	37,135	7,784		655,119	39,416		781	16,809
Jul-20	427	35,137	8,052		668,525	39,881		772	17,074
Aug-20	493	33,192	7,944		643,783	39,281		763	16,334
Sep-20	422	31,027	7,823		636,273	38,944		731	15,975
Oct-20	400	31,866	8,497		654,330	41,439		779	16,451
Nov-20	381	29,627	7,793		587,527	39,873		770	14,929
Dec-20	410	30,260	8,079		608,005	44,541		833	15,930
Jan-21	434	29,373	8,229		620,558	40,220		771	15,209
Feb-21	384	28,165	7,682		575,915	38,023		739	14,233
Mar-21	407	34,915	9,655		737,755	47,200		846	17,330
Apr-21	540	32,710	9,634		723,921	47,468		821	17,284
May-21	520	33,415	10,145		705,825	48,847		773	16,998
Jun-21	482	36,533	10,561		731,710	52,455	1,720	872	17,660
Jul-21	411	35,549	10,197		702,947	49,874	25,725	755	17,209
Aug-21	455	36,601	9,726		665,845	48,355	42,961	758	16,382
Sep-21	413	34,859	9,578		651,193	46,885	52,845	687	16,076
Oct-21	282	32,580	9,742		648,855	45,314	74,946	736	16,376
Nov-21	305	33,002	9,510		615,895	43,512	83,730	689	15,719
Dec-21	333	31,372	9,397		622,660	44,870	92,795	733	15,911
Jan-22	306	30,904	8,963		594,694	38,777	92,198	690	14,788
Feb-22	308	31,198	9,073		597,545	36,967	90,868	697	14,722
Mar-22	425	36,914	10,841		729,221	42,505	114,067	940	18,063
Apr-22	413	30,025	10,524		706,529	42,703	103,700	892	17,854
May-22	341	33,657	10,714		719,271	49,937	85,372	842	18,583
Jun-22	353	31,364	10,906		729,288	59,453	74,702	980	18,880
Jul-22	397	15,427	10,529		682,843	61,537	64,383	958	17,956
Aug-22	406	14,281	11,124		710,322	71,246	65,368	1,013	19,192
Sep-22	395	13,532	10,485	9	668,088	71,221	58,458	924	17,967
Oct-22	376	11,694	10,548	92	656,512	75,603	58,226	810	17,377
Nov-22	357	12,506	10,252	339	629,602	80,797	58,566	479	17,251
Dec-22	331	13,821	10,773	404	633,121	89,659	68,777	544	17,436
Jan-23	353	14,596	11,145	497	668,898	80,629	168,884	484	17,107
Feb-23	328	14,287	10,544	546	644,794	68,454	253,469	485	16,353
Mar-23	376	16,765	12,808	673	763,277	69,556	405,845	443	18,883
Apr-23	372	17,068	11,820	723	720,228	57,675	483,419	340	17,699
May-23	362	19,453	13,097	782	782,163	57,090	552,190	239	19,030
Jun-23	354	19,217	13,922	884	793,091	85,303	454,215	188	19,786

© 2025 Berning P et al. *JAMA Network Open*.

Jul-23	339	18,640	13,521	755	758,334	76,448	428,447	236		19,280
Aug-23	379	20,266	14,862	812	802,175	56,406	481,473	303		21,575
Sep-23	252	19,082	14,359	730	741,666	28,981	420,927	250		21,349
Oct-23	214	20,127	15,486	813	774,684	12,217	429,580	295		23,423
Nov-23	171	19,406	15,208	799	740,487	29,447	410,337	305	686	23,263
Dec-23	146	17,908	14,960	593	722,453	34,231	402,496	312	68,824	22,799
Jan-24	204	34,677	15,366	437	744,494	17,000	410,535	305	183,502	23,383
Feb-24	235	37,700	15,360	398	744,528	6,711	427,720	373	250,132	24,347

© 2025 Berning P et al. *JAMA Network Open*.

eTable 2. Monthly Prescriptions of GLP-1RAs With Weight Loss Effects

Date	MOUNJARO	OZEMPIC	RYBELSUS	VICTOZA
Jul-17				314428
Aug-17				329035
Sep-17				315799
Oct-17				339062
Nov-17				332606
Dec-17				345422
Jan-18		85		346601
Feb-18		1,375		318945
Mar-18		4,460		360075
Apr-18		7,976		351607
May-18		13,843		368748
Jun-18		20,910		355462
Jul-18		27,878		362948
Aug-18		36,847		371543
Sep-18		41,364		339621
Oct-18		56,366		369285
Nov-18		62,491		350398
Dec-18		73,533		356703
Jan-19		92,008		345189
Feb-19		95,129		313139
Mar-19		120,761		343267
Apr-19		136,569		338724
May-19		157,295		345111
Jun-19		167,918		324110
Jul-19		196,212		341994
Aug-19		214,292		336770

© 2025 Berning P et al. *JAMA Network Open*.

Sep-19	218,715		315232
Oct-19	251,912	274	332025
Nov-19	247,293	4,809	307092
Dec-19	279,400	10,276	325315
Jan-20	283,456	17,622	303430
Feb-20	286,676	23,684	283446
Mar-20	348,724	31,420	327049
Apr-20	335,021	32,006	297680
May-20	340,120	33,885	290218
Jun-20	358,024	39,918	294221
Jul-20	369,795	46,471	293578
Aug-20	374,057	50,389	282652
Sep-20	382,057	56,777	278938
Oct-20	405,151	66,790	283703
Nov-20	397,578	68,861	265702
Dec-20	453,082	81,517	291679
Jan-21	434,004	77,173	262258
Feb-21	412,457	76,352	238729
Mar-21	508,231	96,356	277598
Apr-21	500,088	98,170	261365
May-21	511,774	100,937	250801
Jun-21	571,432	113,031	262836
Jul-21	587,862	117,368	255132
Aug-21	621,290	124,021	250041
Sep-21	634,477	126,450	239892
Oct-21	671,650	133,950	236472
Nov-21	690,211	135,108	228685
Dec-21	761,873	151,177	240204
Jan-22	732,186	146,286	218874

© 2025 Berning P et al. *JAMA Network Open*.

Feb-22		718,310	144,940	202129
Mar-22		873,194	172,284	224736
Apr-22		871,352	170,304	206887
May-22		957,563	175,928	205904
Jun-22	14,581	1,055,579	187,691	207902
Jul-22	66,721	1,058,049	183,261	196099
Aug-22	190,181	1,092,120	198,810	203918
Sep-22	325,888	1,122,999	193,046	190581
Oct-22	508,310	1,169,457	198,170	184716
Nov-22	677,253	1,113,981	204,705	183969
Dec-22	700,027	1,247,788	224,824	191985
Jan-23	708,387	1,326,137	231,760	186789
Feb-23	696,826	1,432,867	226,468	170259
Mar-23	789,495	1,716,191	256,111	186995
Apr-23	817,077	1,673,109	235,133	168333
May-23	923,568	1,933,819	253,707	174014
Jun-23	931,957	2,046,620	254,605	168688
Jul-23	829,399	1,913,406	248,947	160257
Aug-23	962,204	2,150,119	263,392	163778
Sep-23	912,793	1,963,534	243,235	143890
Oct-23	1,049,849	1,771,732	255,663	150918
Nov-23	1,129,128	1,990,191	255,295	147292
Dec-23	1,215,333	2,140,395	258,215	141089
Jan-24	1,303,956	1,961,623	249,312	112412
Feb-24	1,214,191	1,959,142	236,715	107167

© 2025 Berning P et al. *JAMA Network Open*.

eTable 3. Monthly Online Searches (per 10 Million Searches) for Obesity Management Drugs

Date	ALLI	CONTRAVE	LOMAIRA	ORLISTAT	PHENTERMINE	SAXENDA	WEGOVY	XENICAL	ZEPBOUND	QSYMIA
Jul-17	123.163013	74.5623023	1.77184104	14.4950106	264.411382	35.4780031		8.148422081		21.89793822
Aug-17	134.241327	70.4566582	2.00210736	13.6397528	256.782992	36.8665179		6.741093672		21.17646101
Sep-17	120.938009	114.165738	1.91964162	12.6210152	239.561659	32.9759843		6.53941781		19.28919177
Oct-17	120.169791	103.66245	2.43463025	14.7658815	244.476383	33.8893726		8.283102856		20.42384013
Nov-17	142.924335	65.637375	3.25854672	12.5454154	239.539717	32.9420298		6.005355164		18.34878083
Dec-17	136.210909	86.0506828	2.4933373	12.9588986	203.963281	27.424402		6.46382418		16.64238496
Jan-18	238.350241	155.185277	3.42488558	15.5572686	248.173807	33.2080422		7.693253377		21.10458339
Feb-18	185.199743	126.286204	2.92813001	15.6138931	265.810011	35.2901139		7.285167866		23.75038647
Mar-18	134.199635	126.925412	2.84771062	14.4561798	295.269595	38.7718598		8.55094243		25.59390635
Apr-18	148.035752	123.084899	3.24895968	15.4392046	294.667748	42.4868258		7.781110154		22.49771143
May-18	129.510905	81.5815732	3.291175	13.7027227	278.964725	40.6066216		6.412483918		21.490659
Jun-18	158.183244	81.862583	3.42942643	13.7736551	291.744136	46.301648		7.151343295		23.8414076
Jul-18	216.266126	66.460309	4.1197644	15.2579477	276.737758	42.0209474		7.75017285		21.66799105
Aug-18	333.225218	59.7431551	3.66733092	14.080218	259.647372	42.4593837		6.745746605		19.44874752
Sep-18	165.001635	80.1673517	3.65649559	13.0359371	235.237094	39.3021755		6.433012315		19.03945842
Oct-18	122.576485	65.9447984	3.79107943	12.8618558	225.484969	43.3102904		6.045424846		19.5866348
Nov-18	117.749501	52.6864799	3.08623879	13.8065466	211.3472	39.473365		5.73059448		16.88327843
Dec-18	160.245527	46.2673981	2.58064251	11.8749774	189.539457	32.2294936		5.714990457		14.49012746
Jan-19	185.018889	48.8123594	3.34712184	13.7245076	243.068292	41.3401029		6.206694399		18.81042973
Feb-19	155.102156	51.943682	4.02904375	14.9031233	266.335639	44.5390761		6.578014778		18.71203972
Mar-19	205.631655	49.0945007	3.71306908	15.8539694	267.387556	43.6917617		6.882038604		19.35985171
Apr-19	139.233578	55.3706093	3.70482015	16.326184	279.048729	44.2375839		5.937878252		18.73462195
May-19	130.546971	52.5543307	4.35549337	14.3057595	268.59585	45.0690371		6.489368029		20.58209983
Jun-19	138.146139	48.3002704	3.48933367	15.2373223	277.218133	44.9528453		5.81425921		19.10453188
Jul-19	114.570064	43.6532402	3.58646396	15.2350991	245.471854	45.0046607		5.087953904		20.72966925
Aug-19	116.330548	41.4114632	3.2060929	15.0972214	253.55358	42.5814566		5.674020437		20.89120769
Sep-19	113.91617	37.5450177	3.74643871	15.5580949	225.497542	38.6527295		5.878217205		18.09695205
Oct-19	108.199549	41.1676323	3.92788778	14.5781499	227.7715	41.8312598		6.354444417		18.64394099
Nov-19	117.758484	39.7986352	3.45807695	13.8754345	204.741462	39.6092786		4.984683729		16.44725872
Dec-19	114.170773	35.5710998	2.77834757	11.977711	197.239618	32.7793026		4.636628142		14.41844647
Jan-20	125.812793	48.5173682	2.88279469	15.7878285	239.844861	44.584824		6.527851921		18.33347272
Feb-20	124.460613	51.4517678	3.46486381	17.5693858	245.442447	50.9166031		6.279935138		19.16629403
Mar-20	106.638936	36.1339457	2.71754965	13.8704694	187.161198	36.0199417		5.66528469		15.88630282
Apr-20	121.990806	39.7862954	3.0650531	15.4347415	194.759675	29.7565931		6.341437054		15.80010353

© 2025 Berning P et al. *JAMA Network Open*.

May-20	140.109121	46.3777601	3.22060673	16.7496618	238.545102	35.3437885		7.598481168	17.47437909
Jun-20	139.725913	51.6103178	4.34477215	17.440219	248.102233	43.3049282		7.442150223	24.31012423
Jul-20	119.646385	46.6365898	3.64690644	14.8373881	224.502729	43.8281872		6.072596352	19.33736945
Aug-20	108.632019	44.2757395	3.60555243	14.9528818	221.932167	42.6386985		5.735296376	19.11755954
Sep-20	136.873738	42.7771428	3.56789838	14.9312117	254.402123	44.2215335		6.221535533	18.95087407
Oct-20	116.754198	48.9048388	3.74814774	16.6004036	234.057119	50.2634502		6.268341459	21.1663211
Nov-20	111.344279	37.1655764	4.11148106	15.9900133	198.529633	43.2793765		7.009331254	16.57973703
Dec-20	117.468382	35.4127998	3.17965668	13.8227353	186.476879	45.4041849		5.777852794	16.26148978
Jan-21	128.111297	39.4408945	2.74480169	17.2590198	218.283661	48.9351399		6.392880194	18.69498656
Feb-21	125.979146	46.6656919	3.20987543	17.7376487	243.579933	56.9468617		7.74821995	23.17414622
Mar-21	123.390987	51.6993816	3.19632222	17.4397711	273.014291	65.5979832		6.962631303	26.54897857
Apr-21	122.290008	56.5456826	4.34729787	19.1325821	278.876663	70.3214285		6.538986977	26.18140715
May-21	127.698802	51.1475551	4.66711318	18.5283821	265.092611	71.1870538	0.70672808	6.812419012	25.39156572
Jun-21	131.579561	54.8378456	3.97074514	16.9562598	283.357234	84.2778784	202.860596	7.873428821	27.79055817
Jul-21	124.39801	44.3996698	4.21166371	17.9593439	265.918503	67.0115038	131.419299	5.344538336	23.73243684
Aug-21	119.692469	39.268337	3.30779069	15.9958324	231.985385	59.6090478	127.585194	7.212103753	25.18486173
Sep-21	103.061771	39.7711607	3.83505103	15.1092226	222.446532	58.2580499	90.669221	5.499349369	21.12589899
Oct-21	106.08392	41.1273762	2.79889668	15.8895947	236.796146	60.4663078	95.4480218	5.737442399	19.6609334
Nov-21	101.094278	34.7789022	3.71283237	15.4750084	204.572073	55.8852747	124.540188	5.480119247	18.1987188
Dec-21	105.207319	31.1533239	2.81735112	13.8911908	189.028412	58.88012	99.2894803	5.691071784	17.13521029
Jan-22	128.074459	42.2748395	3.47361887	16.4315238	219.528795	58.3839219	123.378821	5.895625727	22.69947631
Feb-22	137.277591	52.6269882	3.74946763	19.9726331	272.156847	70.4608617	142.960971	6.030826967	29.89243783
Mar-22	122.877727	56.3983294	4.5561059	20.6754585	296.99006	77.8529173	159.804907	6.483983902	30.76305605
Apr-22	120.926562	53.4151616	4.65161246	20.6383093	270.750131	75.1943188	198.869623	6.965417532	26.30608692
May-22	120.217428	49.3928697	3.83346195	18.5306361	266.677341	86.4123036	166.006207	5.380036603	31.07294695
Jun-22	107.779545	49.6058985	4.5487163	19.5888486	292.168614	108.021212	177.765171	5.94979984	31.07839543
Jul-22	115.071057	55.5190317	5.06855825	18.7974873	277.930071	102.031354	157.39125	6.819758441	27.79912308
Aug-22	125.965344	48.6859808	4.99477191	23.3108181	279.263138	111.314716	147.170869	5.314893675	30.71302151
Sep-22	108.305263	46.2309131	4.13537263	18.2811589	247.410116	110.97805	156.418065	6.250908706	26.27268097
Oct-22	103.508729	49.0594827	4.01282913	16.8921235	256.363178	123.397987	233.993819	5.233319409	27.83881725
Nov-22	101.829672	45.0431419	4.85800992	18.094451	230.971674	125.354218	242.672708	4.086607752	25.22563622
Dec-22	125.989175	46.5339639	4.50086534	15.6477894	229.054775	137.645471	251.270423	4.465068123	24.09093655
Jan-23	129.626373	64.3918449	6.92558403	21.8714385	287.43787	152.158648	540.332248	6.209008133	28.78199491
Feb-23	117.77818	71.4866751	6.13799433	21.9836897	324.061657	125.576489	631.522795	6.256570013	32.33714452
Mar-23	131.669367	69.4967808	6.47944003	23.2801324	331.096948	109.179229	720.800878	6.459794828	32.95077366
Apr-23	128.82978	68.5974337	6.53330543	24.3553607	333.477021	98.4672441	803.820111	5.985329246	35.75881604
May-23	114.708582	69.3597024	7.22085179	24.018369	350.042728	97.2590985	948.255942	6.518772419	37.69869868
Jun-23	113.013496	74.6524511	7.7130621	20.9811444	352.314124	152.176979	926.399065	6.642273	39.80819385

© 2025 Berning P et al. *JAMA Network Open*.

Jul-23	160.618838	65.5739225	6.61832561	20.1680254	319.642224	161.805421	680.879108	5.223925508		33.90139857
Aug-23	109.016297	67.6685934	6.09308926	17.5897116	309.379297	123.667632	701.754359	4.215805333		34.61315606
Sep-23	100.771727	63.9014997	5.78660436	16.8536834	291.079246	107.538865	615.638893	4.214435364		35.24818695
Oct-23	105.016905	62.9245595	5.41774413	18.0238844	274.672089	82.6490118	594.814334	4.967861739	0.970756519	34.50065595
Nov-23	110.092045	60.7999816	5.60449206	17.1715871	264.83993	83.5482674	605.308697	4.550982436	312.665921	32.3212104
Dec-23	102.720473	58.594025	5.74642429	17.3470576	248.951535	74.5268041	569.103436	4.453668244	345.9742429	30.18921884
Jan-24	121.20885	66.6888395	6.64116786	22.0715794	279.094047	85.0568978	625.82817	4.492697934	465.5885389	36.53468375
Feb-24	123.011277	73.2309866	6.96235647	27.6508083	301.827779	79.8625957	636.347703	5.232757517	468.9141427	41.49710034

© 2025 Berning P et al. *JAMA Network Open*.

eTable 4. Monthly Online Searches (per 10 Million Searches) for GLP-1RAs With Weight Loss Effects and Drug Names

Date	MOUNJARO	OZEMPIC	RYBELSUS	VICTOZA	LIRAGLUTIDE	SEMAGLUTIDE	TIRZEPATIDEE
Jul-17				61.634	6.56142519	1.27285521	
Aug-17				69.271	8.28936942	2.05144381	
Sep-17				61.623	9.04359829	1.70947126	
Oct-17				69.620	9.10923427	4.94946123	
Nov-17				73.131	8.62173088	3.01748877	
Dec-17		5.382		60.313	6.68148524	3.56644764	
Jan-18		4.640		78.002	10.6726404	2.80792117	
Feb-18		6.544		79.328	8.8802032	4.0572232	
Mar-18		8.308		78.889	7.94540104	3.49365597	
Apr-18		10.302		74.521	9.03601609	3.36663235	
May-18		12.681		74.326	8.47941813	3.49250001	
Jun-18		17.878		75.970	8.85875694	4.11265963	
Jul-18		22.083		75.621	8.83498271	3.30489334	0.57103995
Aug-18		51.398		72.670	7.81837863	4.60089219	
Sep-18		38.072		60.835	8.45044129	3.2975844	
Oct-18		39.286		64.577	9.68478159	3.24124651	
Nov-18		43.058		59.269	8.61410892	4.18031221	
Dec-18		41.492		55.155	7.37550721	3.61030023	
Jan-19		51.442		67.894	8.44882685	3.89224257	
Feb-19		51.628		65.596	9.39934394	5.29497022	
Mar-19		54.738		62.939	10.7413293	5.53429047	
Apr-19		61.510		65.380	8.92505016	6.18982028	
May-19		60.811		63.695	10.1227663	5.48810049	
Jun-19		60.750		64.502	10.1120977	6.79015333	0.67746221
Jul-19		64.426		60.492	8.83609628	7.72009531	
Aug-19		68.815		57.931	9.79609471	6.14414813	

© 2025 Berning P et al. *JAMA Network Open*.

Sep-19	69.269	5.077	55.047	10.6004887	10.3114743	
Oct-19	78.619	6.066	56.946	8.92759995	8.40020814	0.49529353
Nov-19	74.280	8.348	50.803	8.96157634	9.17584598	
Dec-19	69.739	9.643	49.545	8.00773582	6.59033645	
Jan-20	92.481	14.530	61.486	9.32056639	8.86484308	0.49484499
Feb-20	98.689	16.506	59.167	10.6577018	7.71992326	
Mar-20	74.324	13.728	45.942	8.28212445	5.84942007	0.61044712
Apr-20	61.628	11.011	36.517	8.27840457	5.84155613	
May-20	66.593	13.353	40.882	6.7664854	5.83297573	0.40640949
Jun-20	85.819	16.277	47.985	8.69096818	8.00723325	0.56828276
Jul-20	86.077	18.752	47.292	8.49143763	8.30747333	
Aug-20	82.904	21.914	47.535	8.96149614	8.4323555	0.77320709
Sep-20	88.796	37.457	44.508	9.70619039	9.20282776	0.71493758
Oct-20	94.592	55.498	48.163	9.9215008	10.6090561	0.71234604
Nov-20	87.800	49.422	44.034	8.66569379	9.16775711	0.90518567
Dec-20	96.127	46.599	43.418	7.93450009	10.4738567	1.36004963
Jan-21	108.149	49.237	47.633	9.73347914	9.4691561	0.72417484
Feb-21	144.180	55.678	49.118	11.3826013	110.167057	0.98012325
Mar-21	155.085	50.721	52.472	10.9010542	32.3097293	2.30029342
Apr-21	158.252	53.423	51.119	11.8784461	24.8186014	0.82246907
May-21	155.224	46.070	50.776	11.8578995	32.2544518	1.68541675
Jun-21	216.462	55.847	53.824	12.333024	83.9421998	2.37240028
Jul-21	197.946	55.179	49.033	11.6967127	40.7534138	2.82425796
Aug-21	207.807	49.739	49.342	11.0863534	38.5824096	3.15346234
Sep-21	206.371	48.033	45.947	10.0818344	33.569167	1.82676599
Oct-21	198.641	49.205	47.061	11.2978851	37.6898035	1.91802555
Nov-21	212.923	49.717	44.035	12.3541762	37.4069195	1.55694903
Dec-21	195.109	49.199	38.752	10.0775323	37.0436041	1.88797062
Jan-22	241.440	51.814	45.131	12.7899222	45.3094042	2.54693781

© 2025 Berning P et al. *JAMA Network Open*.

Feb-22		283.635	57.051	50.783	13.1344879	59.1206354	3.71107075
Mar-22		321.208	59.147	51.199	14.6545761	65.3517573	4.13004593
Apr-22		332.925	67.372	45.421	13.3839778	62.9031034	16.7366442
May-22	7.902	400.358	61.183	45.766	12.120183	82.1525693	24.9515864
Jun-22	64.494	456.023	65.661	48.817	13.6368057	96.3356349	151.395292
Jul-22	142.262	488.112	65.941	46.510	12.8559473	101.600823	31.7654449
Aug-22	257.219	557.849	67.619	49.024	14.384099	106.396926	26.5231945
Sep-22	404.438	542.516	69.515	45.436	15.7024773	127.921114	37.8753622
Oct-22	575.467	692.597	69.689	49.428	17.8389519	230.600314	33.8821063
Nov-22	622.403	840.687	83.658	52.995	14.6247357	255.1487	34.5027162
Dec-22	615.018	882.322	84.968	52.701	14.6318595	258.420618	38.9576047
Jan-23	662.848	1417.495	102.148	63.948	16.955703	393.103751	73.8455141
Feb-23	611.721	1626.587	108.429	68.310	18.6032345	436.283517	54.6613816
Mar-23	607.244	1918.281	106.856	64.275	16.2787648	518.57877	64.0651022
Apr-23	666.868	1882.916	111.427	71.064	17.9725219	610.656826	85.1306737
May-23	667.425	2051.615	122.952	76.591	16.3745249	667.731999	95.1870225
Jun-23	717.019	2411.021	143.151	69.660	19.6628361	693.559697	96.3109578
Jul-23	659.491	1914.573	118.602	61.270	19.1551383	597.701038	91.1371181
Aug-23	569.135	1740.749	111.469	61.313	17.5419524	561.114345	99.6407398
Sep-23	500.129	1574.039	87.735	52.980	16.6008916	542.88878	88.3425428
Oct-23	535.742	1693.837	97.325	54.832	17.486769	566.022806	103.910145
Nov-23	673.177	1654.748	98.445	53.172	16.0313971	568.826854	135.558776
Dec-23	610.571	1860.820	96.380	47.901	14.8447752	576.485414	144.390328
Jan-24	655.749	1936.583	100.908	55.180	17.1120765	658.539838	181.312701
Feb-24	653.141	2031.742	88.308	57.136	17.0605024	705.277801	210.496185

© 2025 Berning P et al. *JAMA Network Open*.
