# Supplementary material for: Longitudinal Analysis of Obesity Drug Use and Public Awareness
Source: JAMA Netw Open. 2025 Jan 29;8(1):e2457232. doi: 10.1001/jamanetworkopen.2024.57232 (PMC11780480; doi:10.1001/jamanetworkopen.2024.57232)
Supplement: Supplement 2. — Data Sharing Statement [file jamanetwopen-e2457232-s002.pdf]

Data Sharing Statement

Berning. Longitudinal Analysis of Obesity Drug Use and Public Awareness. *JAMA Netw Open*. Published January 29, 2025. doi:10.1001/jamanetworkopen.2024.57232

Data

Data available: Yes; data are provided in the article and in the supplemental online content file.

Additional Information: Additional information regarding prescriber specialty information are available upon reasonable request from the corresponding author.
